# Understanding and distinguishing three time scale oscillations

**DOI:** 10.1186/1471-2202-15-S1-P54

**Published:** 2014-07-21

**Authors:** Yangyang Wang, Pingyu Nan, Vivien Kirk, Jonathan Rubin

**Affiliations:** 1 Department of Mathematics, University of Pittsburgh, Pittsburgh, PA 15260, USA; 2 Department of Mathematics, University of Auckland, Auckland, 1142, New Zealand

## 

A variety of bursting and spiking patterns arise in models for respiratory neurons [1] and other neurons associated with hormone release [2]. These models often feature quantities evolving on distinct time scales, such as fast voltage and slower ion current activation or inactivation variables. Furthermore, such systems may combine multiple interacting oscillatory mechanisms, such as intrinsic calcium oscillations together with a calcium-dependent, voltage-gated membrane potential oscillation mechanism.

Motivated by the activity observed in these models, the goal of this project is to understand bursting dynamics in three time scale systems. We are particularly interested in knowing how much of the complication in burst patterns results from the presence of three or more timescales in the system and how much is, rather, a reflection of the models being relatively high dimensional nonlinear systems with many parameters. With this motivation, we construct a model consisting of two copies of Morris-Lecar equations with three time scales. By considering two viewpoints within the realm of geometric singular perturbation theory, we explain the mechanisms underlying the bursting dynamics of our model system, making use of computed critical manifold, superslow manifold and bifurcations. To elucidate which characteristics truly represent three time scale features, we investigate certain reductions to two time scales and the parameter dependence of solution features in the three time scale framework.

Our results in this area may be useful for characterizing, developing models of, and analyzing models of experimental data. Moreover, they provide useful information for future modeling studies, where a determination needs to be made about how many time scales to include in a model to represent some given experimental data.
